# Owner misperception of canine body condition persists despite use of a body condition score chart*

**DOI:** 10.1017/jns.2014.25

**Published:** 2014-10-08

**Authors:** Rebekah C. Eastland-Jones, Alexander J. German, Shelley L. Holden, Vincent Biourge, Lucy C. Pickavance

**Affiliations:** 1 Department of Obesity and Endocrinology, Faculty of Health & Life Sciences, Institute of Ageing and Chronic Disease, University of Liverpool, Liverpool, UK; 2 Royal Canin Research Center, Aimargues, France

**Keywords:** Obesity, Dogs, Body composition, BCS, body condition score

## Abstract

Canine obesity is a prevalent disease, but many owners are unaware of it, partly due to misperception of their dog's body shape. Body condition scoring (BCS) is a simple method of assessing body composition, but whether it can reduce owner misperception is unclear. Our aim was to determine the effect of a BCS system on owners' ability to estimate the body condition of their dog. Information from 110 dog owners attending three UK veterinary practices was gathered, by interview, between March and April 2013. First, owners were asked to determine their dog's body condition without guidance, and then reassess it using a five-point BCS chart. Most owners (85/110, 77 %) believed the chart to have improved their ability to estimate the condition of their dog correctly. However, only a weak agreement existed between owner estimates and those of the primary investigator, both with (kappa (*κ*) = 0·28; *P* < 0·001) and without (*κ* = 0·32; *P* < 0·001) the BCS chart. Furthermore, most owners incorrectly estimated their dog's body condition, both with (71/110; 64 %) and without (72/110; 65 %) the chart (*P* = 1·00), with underestimation being most common (with = 63/71, 89 %; without = 66/72, 92 %; *P* = 0·57). Owners of overweight dogs more commonly misperceived their dog's body condition, both with (BCS 1–3: 5/35, 14 %; BCS 4–5: 64/75, 85 %; *P* < 0·001) and without (BCS 1–3: 10/35, 28 %; BCS 4–5: 61/75, 81 %; *P* < 0·001) the BCS chart. Thus, use of a five-point BCS chart does not improve accuracy of owners' perception of their dog's body shape, despite the accompanying perception that it does.

Obesity is a growing concern in dogs^(^^1^^)^, and has numerous adverse health consequences, including increased disease prevalence^(^^2^^)^, decreased lifespan^(^^3^^)^ and metabolic dysfunction^(^^4^^)^. As a result, there is a need for veterinarians to educate owners about what constitutes healthy weight and body condition for their dog. There are various methods for assessing body composition but body condition score (BCS) is the most widely accepted clinical method^(^^5^^)^. BCS systems partition the body composition, continuum into a number of finite categories using the visual and palpable characteristics, and scores correlate well with body fast mass measured by dual-energy X-ray absorptiometry^(^^5^^)^. To aid the assessor, a series of silhouettes are provided that illustrate the visual characteristics for a typical medium-sized dog.

Despite public awareness of the risks of obesity, many overweight people do not believe that they are overweight^(^^6^^–^^8^^)^, and this is a complicating factor for management. Misperception of body weight in third parties has also been recognised, most notably parental misperception of the body shape of their children^(^^9^^)^. Pets are commonly viewed as substitutes for children^(^^10^^)^; unsurprisingly, therefore, some studies have demonstrated that owners often misperceive their dog's body condition^(^^11^^)^. One study demonstrated misperception of body condition by asking owners to compare their dog's shape with images of dogs with varying body conditions^(^^7^^,^^11^^)^. In a second study, dog owners assessed the condition of their dog by relating it to five-word descriptions, with no additional guidance given^(^^7^^)^. However, neither study stated whether or not BCS systems were used when scoring pets. As a result, the aims of the present study were to determine the degree to which dog owners misperceive body condition, and whether or not this could be improved using a BCS chart.

## Experimental methods

### Study design

The study followed a within-subject, cross-sectional design by interview of clients attending veterinary practices. All methods adhered to animal ethics guidelines set by the University of Liverpool and were approved by the University of Liverpool Research Ethics Committee.

### Participants

The study was conducted at three centres: The University of Liverpool Small Animal Teaching Hospital, Neston, UK; The University Veterinary Practice, Liverpool, UK; and the Cornyard Veterinary Practice, Oxon, UK between March and April 2013. During this period, the primary study investigator attended each centre, during normal working hours, and conducted interviews with dog owners. While waiting to see the veterinarian, owners were approached and, if they agreed to participate, then were given an information sheet to read and consent form to sign (see Supplementary Appendix 1). For an individual to be capable of giving informed consent, they must be able to understand and retain a piece of information, use it in a decision-making process, and then communicate their decision to the person requesting consent^(^^12^^)^. For this study, prior to proceeding, owners were asked questions about their dog's behaviour (as determined by the Shepherd's Ladder of Aggression)^(^^13^^)^. Not only did this confirm their capacity to consent, it also confirmed the animal's suitability. Owners then signed a consent form and the study began.

### Eligibility criteria

All dogs that attended the practice on the days that interviews were performed were eligible for inclusion. Additional eligibility criteria included owner consent to being interviewed, and the owner indicating that their dog was not aggressive (to ensure that a BCS examination could be conducted without the risk of injury to owner or personnel).

### Body condition scoring

First, the primary investigator assigned each dog a score using a commercially available five-point BCS system (Supplementary figure). To avoid bias, the owner was not informed of their dog's score until after the interview had been completed.

### Interviews

Owners' perception of canine body condition and awareness of obesity were assessed using face-to-face interviews, consisting of a series of both open- and closed-ended questions, and lasting for approximately 5 min (see Supplementary Appendix 2). Only one person responded to the questions and, therefore, accompanying friends and family were not asked to contribute.

First, the study investigator asked the owner to assess their dog's body condition without any guidance. For this, owners were asked to choose the term that best applied to their dog, by selecting one of the following options: ‘very thin’, ‘thin’, ‘ideal weight’, ‘overweight’ or ‘markedly obese’. These phrases were identical to those used in a five-point BCS chart. Next, owners were given a copy of the five-point BCS chart, and the investigator guided them on how to use it (but without specifically referring to the body condition of the owner's dog). Owners then used the chart to assess the BCS of their dog, using a combination of visual inspection and palpation. Open-ended questions were then used to determine owner's awareness of canine obesity, awareness of BCS and their opinions on the BCS chart. Following interview, owners were given a debriefing sheet to provide more information about the study, and to discuss the significance of the dog's true BCS.

### Data collection and statistical analyses

Qualitative and quantitative analyses of interview questionnaire responses from dog owners were used to determine how well owners perceived the body condition of their dog, the usefulness of the BCS chart and which aspects of the chart should be improved. Statistical analyses were conducted using computer software (StatsDirect, version 2.6.8, StatsDirect Ltd). Since questionnaire responses comprised categorical data, non-parametric methods were chosen. The prevalence of misperception was calculated as the proportion of owners who incorrectly assessed their dog's body condition. For this, the investigator's assessment of canine body condition was assumed to be the ‘gold standard’. Fisher's exact test was used to examine the relationship between the misperception of owners of dogs that attracted conflicting investigator-classified scores (i.e. overweight *v.* normal or underweight) and misperception with and without the BCS chart. Agreement between the investigator and participant assessment of canine body condition was assessed using weighted kappa (*κ*) analysis. The level of statistical significance was set at *P* < 0·05 for two-sided analyses.

## Results

### Dogs

In total, 110 dogs were enrolled, fifty nine of which were male, and fifty one were female. Median age was 6 years (range 1–16 years), and thirty-seven breeds were represented, including Labrador retriever (twelve dogs), crossbred (twenty dogs), Boxer (eight dogs), West Highland Terrier (seven dogs) and ‘other’ (sixty-three dogs). Median BCS determined by the primary study investigator was 4/5 (range 1/5–5/5).

### Owner misperception of body condition

The results of BCS by both the study investigator and owners are given in Table 1. Without the BCS chart, owner misperception was common, with 72/110 (66 %) of scores differing from those of the primary investigator. When the BCS chart was subsequently used, a similar degree of owner misperception persisted (*P* = 1·000), with different scores being assigned in 71/110 (65 %) of dogs. Only 17/110 (15 %) owners changed their scores, with twelve of seventeen changing to a correct score and five of seventeen changing to an incorrect score. Underestimating BCS was the most common, and use of the chart did not influence this (without the chart: 63/110 (57 %) underestimated; with the chart 66/110 (60 %) underestimated; *P* = 0·684). Overestimation did occur, but this was less common, and again there was no difference whether or not the BCS chart was used (without the chart 9/110 (8 %) of dogs overestimated compared with the investigator; with the chart 5/110 (4 %) of dogs overestimated compared with the investigator; *P* = 0·287).

**Table 1.** Body condition scores (BCS) assigned by the study investigator and owners

		Owner	
BCS category	Investigator	Without chart	With chart
1	2 (2 %)	0 (0 %)	2 (2 %)
2	8 (7 %)	7 (6 %)	7 (6 %)
3	25 (23 %)	65 (59 %)	67 (61 %)
4	38 (34 %)	37 (34 %)	32 (29 %)
5	37 (34 %)	1 (1 %)	2 (2 %)

**Fig. 1.**
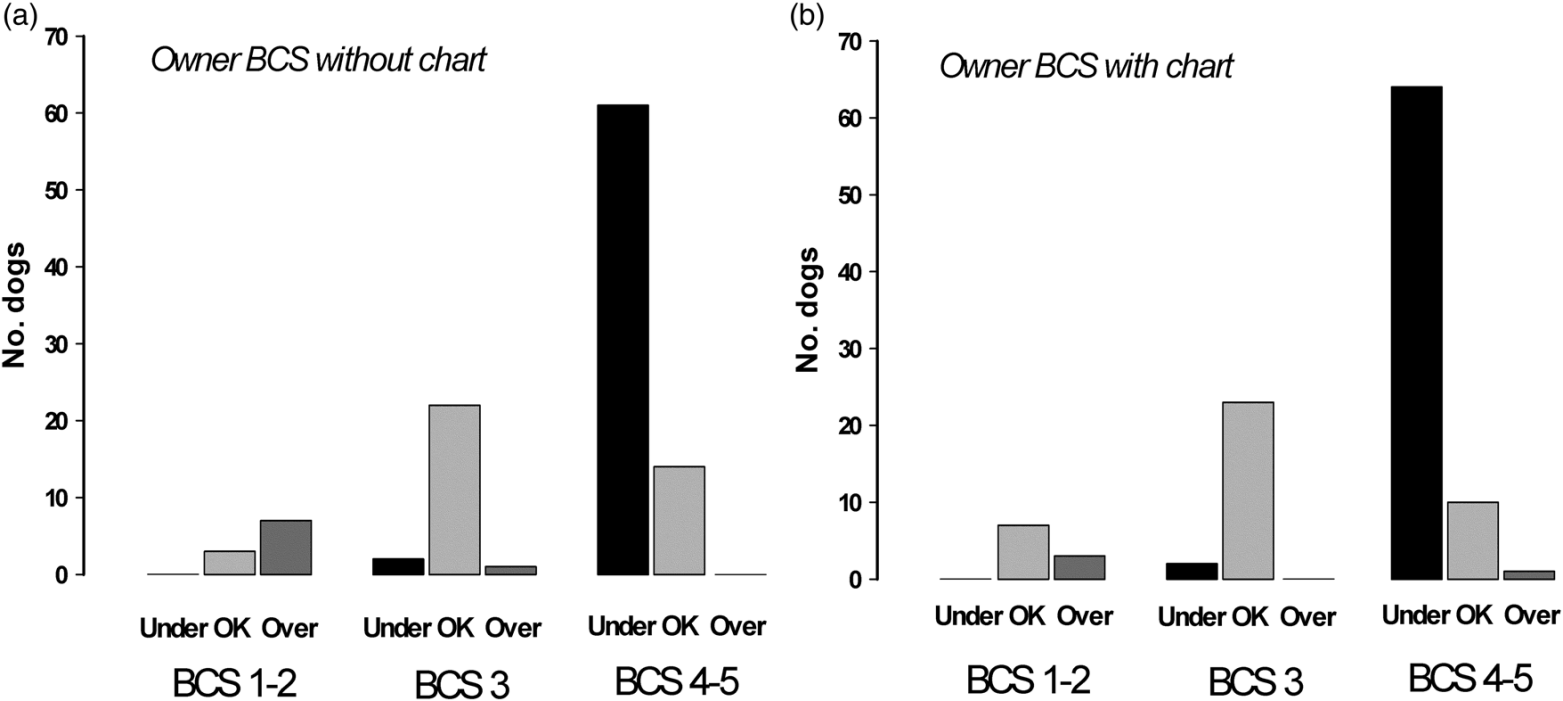
Comparison of body condition scores (BCS) of owners compared with the primary study investigator, either without (a) or with reference (b) to a BCS chart. Dogs are categorised according to the BCS assigned by the primary investigator, as BCS 1–2/5, BCS 3/5 and BCS 4–5/5. For each category, owner scores are depicted as an underestimate (under, black), overestimate (over, dark grey) or exact (OK, light grey). Agreement between the study investigator and owners was poor for overweight dogs, with BCS being underestimated in most cases.

When misperception was stratified by body condition (i.e. underweight, BCS 1–2/5; ideal weight, BCS 3/5; overweight BCS 4–5/5), there were differences between how commonly owners incorrectly scored their dog (Fig. 1) ‘When misperception was stratified by…scored their dog (Fig. 1)’. Without the chart, owners underestimated sixty-one (81 %) of the seventy-five overweight dogs and a similar number (sixty four, 85 %) were underestimated when the BCS chart was subsequently used. Owners overestimated the condition of underweight in seven of ten (70 %) dogs without the chart and in three of ten (30 %) when they used the chart. In contrast, of the twenty-five dogs in ideal body condition, owners incorrectly scored only three (12 %) and one (8 %) without and with the BCS chart, respectively.

The *κ* analysis showed good agreement between owner scores selected without and with the use of the chart (*κ* = 0·751; *P* < 0·001). However, only fair agreement was seen between the investigator and owner scores, both without the chart (*κ* = 0·277; *P* < 0·001), and when it was subsequently used (weighted *κ* = 0·317; *P* < 0·001). To explore the owner misperception further, the *κ* analysis was used to assess agreement with investigator scores separately in overweight (BCS 4–5/5 as determined by the primary investigator) and underweight (BCS 1–2/5 as determined by the primary investigator) dogs. For overweight dogs, there was no significant agreement regardless of whether the chart was used (without the chart: *κ* = 0·014, *P* = 0·154; with the chart: *κ* = 0·000, *P* = 0·492). In contrast, for underweight dogs, no agreement was seen without the BCS chart (*κ* = −0·167; *P* = 0·943), but moderate agreement was seen when the chart was used (*κ* = 0·516; *P* < 0·001).

### Owners' opinions on using the body condition score chart

In total, 90 % (99/110) of owners were aware of the health implications of obesity, but 93 % (102/110) were not aware of the existence of BCS charts or how to use them. In total, 77 % (85/110) of owners believed that the BCS chart had improved their ability to estimate the body condition of their dog. However, when comparing owners who believed the BCS chart had helped them, with those who had not, there was no difference in the proportion of scores that improved after using the chart (seven of eighty five (8 %) for those believing the chart had made a difference *v*. two of twenty five (8 %), for those believing the chart made no difference; *P* = 1·000).

When participants were asked which aspects of the chart were most helpful, nearly half (51/110; 46 %) mentioned the silhouette images. When participants were asked which aspects of the chart could be improved, most (82/110, 75 %) did not believe any change was necessary. Of the remaining owners, 22/110 (20 %) suggested improvements to the silhouette images, including using breed-specific silhouettes, making the images more detailed, using real photographs or making a greater distinction between shapes assigned to the different categories.

## Discussion

In agreement with recent studies^(^^7^^,^^14^^)^, the present study has demonstrated that most owners misperceive the body condition of their dog. However, the present study is novel in that it has also demonstrated that, for overweight dogs, the owner misperception persists even when a BCS chart is used. Therefore, owners cannot be relied on to estimate their dog's body condition accurately. The present study has also highlighted the need for greater emphasis on education of the public regarding companion animal obesity. In this respect, most of the owners interviewed had no prior knowledge of BCS charts and how to use them. This might be due to the fact that veterinarians do not commonly weigh dogs or estimate their body condition^(^^15^^)^, and then do not record in computers that a dog is overweight or obese^(^^16^^)^. Tackling owner misperception of body condition is arguably one way whereby veterinarians could improve owner awareness of what constitutes a healthy weight and the need to avoid obesity.

In the present study, owners tended to ‘normalise’ their dog's body condition, with condition being under- and overestimated in overweight and underweight dogs, respectively. However, this tendency was more pronounced for overweight dogs, since owners underestimated body condition irrespective of whether or not they used a BCS chart. This is similar to the parental misperception of body shape, which occurs more often when the child is overweight^(^^9^^)^, suggesting that owners might be unwilling to accept that their dog is obese, again similar to parents attitudes towards childhood obesity^(^^17^^)^. Obesity is publicised negatively by the media, and this can affect the willingness of parents to admit that their child is overweight^(^^18^^)^. Negative portrayals of obesity in the veterinary and animal press might have a similar effect, as suggested by a recent study which revealed that most owners do not believe obesity to be a substantial health concern for dogs^(^^19^^)^.

Despite the majority (90 %) of participants stating that they were aware of the health risks associated with canine obesity in the present study, a third of participating dogs were classified as obese. This again suggests that owners are reluctant to acknowledge that their own animal is obese. In a previous study, a major hurdle to enrolling dogs on weight loss programmes was that owners were unwilling to deny their pet food^(^^20^^)^. A further study suggested that dog owners preferred to accept the adverse health implications associated with obesity rather than change of feeding habits^(^^11^^)^. Therefore there is a clear need to improve owner education regarding what constitutes a healthy canine body shape and why this is important to maximise their pet's health and wellbeing. Most participants believed that the BCS chart made it easier for them to determine the body condition of their pet even though, in reality, it had not. This might be because owners had a preconceived idea of their pet's body condition and simply used the BCS chart to confirm their belief. The fact that changing score did not improve accuracy of owner scores suggests that training is required before such systems can be used with any reliability.

Almost half (46 %) of the participants identified the image as the most helpful aspect of the BCS chart. That said, determining body condition from images is subjective, and perception of visual stimuli is dependent on experience, knowledge, cognition or understanding of symbols by the individual^(^^21^^)^. Indeed, many owners suggested for improving the silhouette images, for instance, using photographs, providing more detailed drawings and showing dogs with different body shapes. The latter is not surprising; most current systems use a medium-sized breed shape, akin to the Labrador retriever, and it may be difficult to determine the condition of breeds with very different body structures. For example, the shape of a Greyhound or Bulldog differs greatly in morphology, and the silhouette of a Labrador retriever is, arguably, not helpful. In future, those developing BCS systems should consider making them more breed specific.

The present study has a number of limitations, which should be considered when interpreting the findings. First, since dogs and owners were recruited from a veterinary-visiting population, most had underlying medical conditions, and this might have influenced both the body condition of the dog and the owners' perception of condition. Furthermore, the choice of a veterinary-visiting population might have inadvertently selected for more responsible and knowledgeable pet owners, and increased the likelihood that owners were aware of issues regarding body condition and weight status. A second limitation was the fact that only one investigator assessed body condition, and her scores might have been susceptible to observer error^(^^22^^)^. However, a previous work has suggested that different assessors typically under- or overestimate BCS by the same magnitude when assessing body condition^(^^23^^)^. Therefore use of a single investigator would have led to only a limited variation in her scores. Concerns over inaccuracies in investigator BCS scores were further circumvented by the use of *κ* analysis, which simply compares the results between investigators without the need for pre-determining which is correct. A final concern was the reliability of information obtained from the interviews^(^^11^^)^. Although it is assumed that participants answer truthfully, this may not be the case, especially with questions on sensitive topics. Indeed, the more controversial the content of a question, the greater the likelihood of an untruthful answer being given^(^^24^^)^.

## Conclusion

In summary, this study indicates that, despite the fact that owners are aware of the health consequences of canine obesity, they frequently misperceive the body condition of their own dogs. Furthermore, for overweight dogs, owner misperception persists, whether or not a BCS chart is used. Additional studies are needed to determine the reasons for this misperception, and to identify methods by which veterinarians can better educate owners regarding optimal body shape and the need to identify unwanted weight gain.

## Supplementary material

The supplementary material for this article can be found at http://www.journals.cambridge.org/jns
